# Mania a Potu

**Published:** 1887-05

**Authors:** Clarence C. Howard

**Affiliations:** New York, N. Y.


### MANIA A POTU.

Clarence C. Howard, M. D., New York, N. Y.

Case I.—Mr. F., admitted to the "New York Christian Home for Intemperate Men" March 25th, 1886, he having previously arranged that his physician, a homœopathist (?), should attend him. Eight days later, when I saw him, I obtained the following history: This was his eighth attack of delirium tremens, and his physician, after having prescribed thirty grains of Chloral Hydrate twice a day, also a dark-colored liquid in two glasses of water, concluded he had done all that medical science was capable of, and had abandoned the case as hopeless. The symptoms were so masked by this barbarous, inhuman treatment, that I was unable to prescribe until the next day, when the following condition presented itself: Delirious talking, thinks that members of his family have been murdered; sometimes thinks they have been burnt, and that their charred remains are in the room with him; sometimes that they were eaten by vermin; great fear that he will become insane, or that people will think him so.

There was a profuse perspiration about the head and face, and the legs and feet were cold and clammy. He was extremely nervous and easily frightened; sometimes would crouch in the corner of the room and pull the bed-clothing over him.

One powder of Calc. carb.$^{107m}$ (Fincke). There was no improvement until the evening of the 9th of April, when he fell into a deep sleep which lasted until about eleven o'clock the next morning. He awakened perfectly rational and remained so until about eleven P. M., when he had a return of the delirium, which continued for twenty-four hours, when he again fell asleep, sleeping for about twelve hours. Upon awaking his delusions and hallucinations had entirely left. He had but the *one* dose.

CASE II.—Mr. A., age thirty-seven, admitted to the "New York Christian Home for Intemperate Men" April 14th, 1886. Having been on a protracted spree for three months, developed mania a potu two days later. Symptoms: Delirious talking; amorous talking, sees beautiful women in a nude state; very merry; beautiful visions, changing to horrible; picking at the bed-clothes. One powder Cannabis Indica $^{5m}$. (Fincke). Slept all the night of the 18th; awakened perfectly rational the next morning. No return of symptoms.

CASE III.—Mr. H., age thirty, admitted to the "New York Christian Home for Intemperate Men" June 8th, 1886. Mania a potu developed June 10th. Symptoms: Delirious talking, pupils dilated, eyes congested, constantly smiling; desire to escape from the room; cannot realize where he is; ugly moods, tries to bite the nurse or the spoon with which he is fed. Bell.$^{mm}$ (Fincke), one powder. Fell asleep the night of June 11th; June 12th perfectly rational. No further trouble.

